# Association between the *STK15* F31I Polymorphism and Cancer Susceptibility: A Meta-Analysis Involving 43,626 Subjects

**DOI:** 10.1371/journal.pone.0082790

**Published:** 2013-12-13

**Authors:** Weifeng Tang, Hao Qiu, Hao Ding, Bin Sun, Lixin Wang, Jun Yin, Haiyong Gu

**Affiliations:** 1 Department of Cardiothoracic Surgery, Affiliated People’s Hospital of Jiangsu University, Zhenjiang, China; 2 Department of Microbiology and Immunology, Medical School of Southeast University, Nanjing, China; 3 Department of Respiratory Disease, Affiliated People’s Hospital of Jiangsu University, Zhenjiang, China; Sapporo Medical University, Japan

## Abstract

The association between the Serine/threonine kinase 15 (STK15) F31I polymorphism (rs2273535) and cancer susceptibility remains controversial. To further investigate this potential relationship, we conducted a comprehensive meta-analysis of 27 published studies involving a total of 19,267 multiple cancer cases and 24,359 controls. Our results indicate statistical evidence of an association between the *STK15* F31I polymorphism and the increased risk of overall cancer in four genetic models: AA vs. TA+TT, AA vs. TT, AA vs. TA, and A vs. T. In a stratified analysis by cancer type, there was an increased risk of breast cancer in four genetic models: AA vs. TA+TT, AA vs. TT, AA vs. TA, and A vs. T, as well as esophageal cancer in two genetic models: AA vs. TA+TT and AA vs. TA. In a stratified analysis by ethnicity, there was a significant increase in cancer risk among Asians, but not Caucasians, in four genetic models: AA vs. TA+TT, AA vs. TT, AA vs. TA and A vs. T. In addition, a stratified analysis by ethnicity in the breast cancer subgroup revealed a significant increase in cancer risk among Asians in two genetic models: AA vs. TA+TT and AA vs. TT, as well as among Caucasians in one genetic model: AA vs. TA. In summary, this meta-analysis demonstrates that the *STK15* F31I polymorphism may be a risk factor for cancer.

## Introduction

 Cancer is a complex disease that results from interactions between multiple genetic and environmental factors [1-3]. A characteristic of cancer is genetic instability, which can be caused by transgenation and acquired aneuploidy [4]. Genetic instability mostly occurs at the chromosomal level, including losses and gains of whole or large portions of chromosomes [5]. Chromosomal segregation is accomplished by the mitotic spindle, which links whole chromosomes to opposite poles of the cell, and segregates the duplicated DNA equally into two daughter cells [6]. In mammalian cells, centrosomes are the major microtubule organizing centers (MTOC) and play a vital role in symmetrical mitotic spindle formation and mitosis. Serine/threonine kinase 15 (STK15), a centrosome**-**localized serine/threonine kinase, acts as a critical regulator of mitotic centrosome maturation and spindle assembly. It has a particular role in G2 to M phase, primarily through its phosphorylation functions, and plays an important role in the development and progression of cancer malignancy [7].

 A non-synonymous single nucleotide polymorphism (SNP) of *STK15*, the F31I polymorphism (rs2273535), has been identified in the coding region of *STK15*. The *STK15* F31I polymorphism (91 T→A), a SNP in exon 3 of *STK15*, encodes a phenylalanine→isoleucine substitution at amino acid residue 31 (F31I) [8]. In recent years, the F31I polymorphism has been intensely investigated for its association with the risk of multiple cancers. Many studies have indicated that the *STK15* F31I polymorphism is a general low penetrance susceptibility gene in a number of cancers, particularly breast, colorectal, and esophageal cancer [9-11]. However, results from these studies remain inconsistent, perhaps due to small sample size limitations, ethnic diversity in allele frequencies, and publication bias. Therefore, to confirm the role of the *STK15* F31I polymorphism in tumorigenesis, we conducted a comprehensive meta-analysis on eligible case-control studies published to date. To the best of our knowledge, this is the most comprehensive meta-analysis regarding the *STK15* F31I polymorphism and its association with cancer risk.

## Materials and Methods

This meta-analysis is reported according to the Preferred Reporting Items for Systematic Reviews and Meta-analyses (PRISMA) guideline (Table **S1**. PRISMA checklist) [12]. 

### Search Strategy

 Genetic association articles published on cancer and the *STK15* F31I polymorphism, up to May 29, 2013, were investigated by searching PubMed, EMBASE, CBM (Chinese BioMedical Disc) and CNKI (Chinese National Knowledge Infrastructure) with combinations of the following terms: "stk15", "Aurora-A", "BTAK", "AIKI", "polymorphism", "SNP", "mutation", "carcinoma", "cancer", "neoplasm", and "malignance". In addition, the publication language was restricted to English and Chinese. All bibliographies listed in these studies and published reviews were checked for original and relevant studies.

### Inclusion and Exclusion Criteria

 Eligible studies had to meet the following criteria: 1) evaluated the *STK15* F31I polymorphism and cancer risk, 2) designed as a case-control study, 3) provided data on genotype or allele frequency in case groups and control groups, 4) provided the genotyping method and ethnicity, and 5) control genotype distributions consistent with Hardy-Weinberg equilibrium (HWE). Exclusion criteria included the following: 1) overlapping data, 2) not case-control studies, and 3) review publication.

### Data Extraction

 Information from all eligible publications was carefully and independently extracted through three reviewers (W. Tang, H. Qiu, and H. Ding). In case of conflicting evaluations, differences were resolved by further discussion among all reviewers. For each included study the following data was extracted: first author, cancer type, year of publication, country, ethnicity of study subjects, number of cases and controls, genotype method, allele and genotype frequency, and HWE in controls.

### Statistical Analysis

 Deviation from the HWE among the controls was evaluated for each single study using an internet-based HWE calculator (http://ihg.gsf.de/cgi-bin/hw/hwa1.pl). The crude odds ratio (OR) with the corresponding 95% confidence intervals (95% CI) was used to measure the strength of the association between the *STK15* F31I polymorphism and cancer risk. The significance of the pooled OR was assessed using the Z-test and *P*-value (two-tailed), and *P*<0.05 was considered statistically significant. In our study, a Chi-square-based I^2^ test was used to check potential heterogeneity among studies; I^2^<25% indicated low heterogeneity, 25%≤I^2^≤50% indicated moderate heterogeneity, and I^2^>50% indicated large heterogeneity [13]. The heterogeneity was considered statistically significant at I^2^>50% or *P*<0.10. If heterogeneity existed, the pooled ORs were calculated according to the random-effects model (the DerSimonian–Laird method) or the fixed-effects model was used (the Mantel–Haenszel method). Subgroup analyses were conducted according to ethnicity and cancer type to measure ethnicity-specific and cancer type-specific effects (any cancer type evaluated by less than three individual case-control studies was combined into "other cancers"). Sensitivity analysis was also carried out to determine whether any excluded studies affected the stability of our results. Galbraith radial plot and further stratified analyses were used to analyze the source the heterogeneity. In our studies, the funnel plot and Egger’s test were used to assess potential publication bias, which was measured by visual inspection of an asymmetric plot. In addition, for the interpretation of Egger’s test, statistical significance was defined as *P*<0.05. Statistical analyses were performed using STATA (v12.0) statistical software.

## Results

### Characteristics

**Figure 1:**
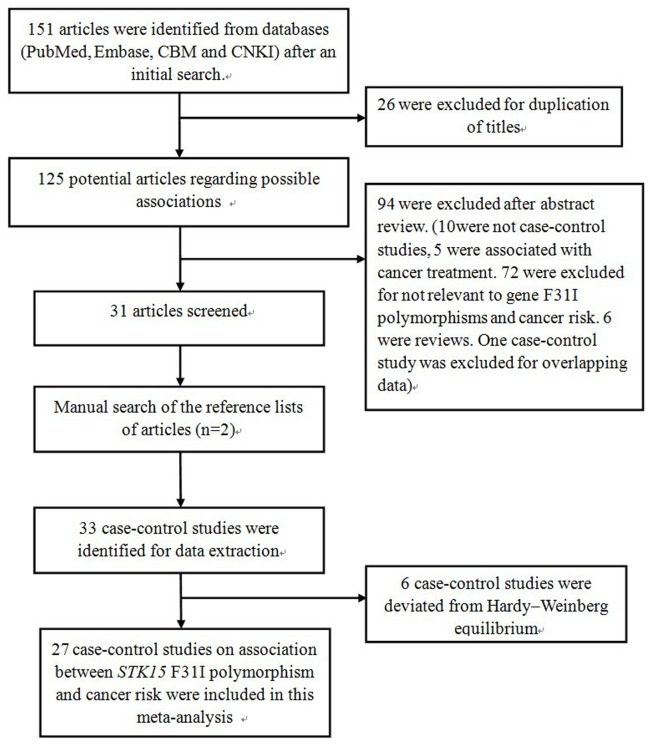
Flow diagram of articles selection process for *STK15* F31I polymorphism and cancer risk meta-analysis.

 After an initial search, a total of 151 published articles relevant to the topic were identified from databases (PubMed, Embase, CBM and CNKI). With additional filters, 120 of these articles were excluded (26 for duplication of titles, 10 for not being case**-**control studies, five for an association with cancer treatment, 72 for irrelevance to gene polymorphisms and cancer, six reviews and one case-control study for overlapping data). After this step, 31 qualified and original papers fit the inclusion criteria. After a manual search of the bibliography lists from retrieved articles, another two articles were included (Figure **1**). Afterwards, six case-control studies were excluded because the number of genotypes in the control group statistically deviated from HWE. Overall, 27 total case-control studies on the association between the *STK15* F31I polymorphism and cancer risk were recruited in this meta-analysis. Among the 27 case-control studies, ten investigated breast cancer [8,9,14-21], four investigated colorectal cancer [10,22-24], and three investigated esophageal cancer [11,25,26]. The other studies investigated gastric cancer, lung cancer, renal cell carcinoma, bladder cancer, glioblastoma, hepatocellular carcinoma, and ovarian cancer [27-36]. As for subjects in these studies, 11 were Asian [9,11,19-21,23,25-29] and 16 were Caucasian [8,10,14-18,22,24,30-36]. Characteristics of populations and cancer types in each individual study recruited in the meta-analysis are listed in Table **1**. The distribution of the *STK15* F31I polymorphism and allele among patients and controls is listed in Table **2**. Results of the meta-analysis from different comparative genetic models are summarized in Table **3**, Table **4**, and Table **5**.

**Table 1:** Characteristics of populations and cancer types of the individual studies included in the meta-analysis.

Study	Year	Ethnicity	Country	Cancer type	Sample size (case/control)	Genotype method
Sang et al.	2012	Asians	China	esopheal cancer	380/380	MALDI-TOF MS
Ruan et al.	2011	Asians	China	breast cancer	1334/1568	TaqMan
Navaratne et al.	2010	Caucasians	USA	glioblastoma	96/93	PCR-RFLP
Akkiz et al.	2010	Caucasians	Turkey	hepatocellular carcinoma	128/128	PCR-RFLP
Song et al.	2010	Asians	China	bladder cancer	60/60	PCR-RFLP
Chen et al.	2009	Asians	China	esopheal cancer	188/324	PCR-RFLP
MARIE-GENICA	2009	Caucasians	German	breast cancer	3136/5466	MALDI-TOF MS
Ricketts et al.	2009	Caucasians	Polish	renal cell carcinoma	328/311	MLPA
Dogan et al.	2008	Caucasians	Turkey	lung Cancer	102/102	Direct sequencing
Chen et al.	2007	Caucasians	USA	colorectal cancer	60/65	Direct sequencing
Wang et al.	2007	Caucasians	USA	lung cancer	1518/1518	TaqMan
Vidarsdottir et al.	2007	Caucasians	Iceland	breast cancer	759/653	TaqMan
Tchatchou et al.	2007	Caucasians	German	breast cancer	727/819	TaqMan
Hammerschmied et al.	2007	Caucasians	German;USA	renal cell carcinoma	156/158	PCR-RFLP
Webb et al.	2006	Caucasians	UK	colorectal cancer	2558/2680	Illuminasentric bead array
Fletcher et al.	2006	Caucasians	UK	breast cancer	507/875	PCR-RFLP
Zhang et al.	2006	Asians	China	colorectal cancer	283/283	PCR-RFLP
Cox. et al.	2006	Caucasians	USA	breast cancer	1259/1742	TaqMan
Ju et al.	2006	Asians	Korea	gastric cancer	501/427	MALDI-TOF MS
Chen et al.	2005	Asians	China	gastric cancer	68/75	PCR-RFLP
Hienonen et al.	2005	Caucasians	Finland	colorectal cancer	235/94	Direct sequencing
Lo et al.	2005	Asians	China(Taiwan)	breast cancer	709/1972	TaqMan
DiCioccio et al.	2004	Caucasians	UK;Denmark;USA	ovarian Cancer	1821/2467	TaqMan
Sun et al.	2004	Asians	China	breast cancer	520/520	PCR-RFLP
Egan et al.	2004	Caucasians	USA	breast cancer	940/830	Direct sequencing
Miao et al.	2004	Asians	China	esopheal cancer	656/656	PCR-RFLP
Dai et al.	2004	Asians	China	breast cancer	1193/1310	TaqMan

MALDI–TOF MS: Matrix-Assisted Laser Desorption/Ionization Time of Flight Mass Spectrometry

PCR-RFLP: polymerase chain reaction-restriction fragment length polymorphism

MLPA: Multiplex Ligation Dependent Probe Amplification

**Table 2:** Distribution of *stk15* *F31I* polymorphisms genotype and allele among multiple cancer patients and controls.

	Case			Control			Case		Control		HWE
	AA	TA	TT	AA	TA	TT	A	T	A	T	
Sang et al.	46	161	173	39	188	153	253	507	266	494	Yes
Ruan et al.	167	568	599	161	691	716	902	1766	1013	2123	Yes
Navaratne et al.	4	33	59	6	33	54	41	151	45	141	Yes
Akkiz et al.	4	47	77	2	27	99	55	201	31	225	Yes
Song et al.	33	15	12	18	25	17	81	39	61	59	Yes
Chen et al.	66	79	43	118	168	38	211	165	404	244	Yes
MARIE-GENICA	167	1096	1873	249	1927	3290	1430	4842	2425	8507	Yes
Ricketts et al.	207	105	16	171	122	18	519	137	464	158	Yes
Dogan et al.	6	38	58	3	40	59	50	154	46	158	Yes
Chen et al.	3	13	44	6	21	38	19	101	33	97	Yes
Wang et al.	36	373	692	51	320	594	445	1757	422	1508	Yes
Vidarsdottir et al.	42	288	429	21	231	401	372	1146	273	1033	Yes
Tchatchou et al.	433	257	37	485	287	47	1123	331	1257	381	Yes
Hammerschmied et al.	7	57	92	12	65	81	71	241	89	227	Yes
Webb et al.	114	880	1564	125	888	1667	1108	4008	1138	4222	Yes
Fletcher et al.	18	154	335	48	280	547	190	824	376	1374	Yes
Zhang et al.	142	111	30	104	137	42	395	171	345	221	Yes
Cox. et al.	66	401	774	65	571	1075	533	1949	701	2721	Yes
Ju et al.	211	215	75	179	190	58	637	365	548	306	Yes
Chen et al.	36	27	5	33	32	10	99	37	98	52	Yes
Hienonen et al.	19	94	122	5	43	46	132	338	53	135	Yes
Lo et al.	348	288	71	886	887	196	984	430	2659	1279	Yes
DiCioccio et al.	71	502	821	99	649	1213	644	2144	847	3075	Yes
Sun et al.	256	214	50	192	262	66	726	314	646	394	Yes
Egan et al.	50	331	559	31	283	516	431	1449	345	1315	Yes
Miao et al.	308	290	58	249	316	91	906	406	814	498	Yes
Dai et al.	490	491	121	534	503	149	1471	733	1571	801	Yes

HWE: Hardy–Weinberg equilibrium.

**Table 3:** Summary of results of the meta-analysis from different comparative genetic models in the subgroup analysis by ethnicity.

Polymorphism	Genetic comparison	Population	OR(95%CI)	*P*	Test of heterogeneity		Model
					*p* -Value	I^2^	
	AA+TA vs. TT	All	1.04(0.97-1.12)	0.265	0.002	50.1%	R
		Asians	1.07(0.89-1.28)	0.482	0.001	65.6%	R
		Caucasians	1.04(0.97-1.11)	0.305	0.084	34.8%	R
	AA vs. TA+TT	All	**1.18(1.06-1.31)**	**0.002**	0.000	56.2%	R
		Asians	**1.27(1.10-1.47)**	**0.001**	0.002	64.8%	R
		Caucasians	1.08(0.93-1.26)	0.310	0.026	45.3%	R
	AA vs. TT	All	**1.16(1.01-1.32)**	**0.035**	0.000	55.7%	R
		Asians	**1.26(1.01-1.56)**	**0.039**	0.001	66.5%	R
*STK15* F31I		Caucasians	1.08(0.91-1.28)	0.388	0.031	43.9%	R
	TA vs. TT	All	1.01(0.95-1.08)	0.745	0.028	37.2%	R
		Asians	0.96(0.81-1.13)	0.628	0.015	54.6%	R
		Caucasians	1.03(0.98-1.08)	0.224	0.247	18.0%	F
	AA vs. TA	All	**1.18(1.06-1.30)**	**0.001**	0.003	48.4%	R
		Asians	**1.28(1.12-1.47)**	**0.000**	0.010	57.0%	R
		Caucasians	1.07(0.93-1.23)	0.342	0.081	35.2%	R
	A vs. T	All	**1.08(1.01-1.14)**	**0.015**	0.000	64.4%	R
		Asians	**1.14(1.02-1.28)**	**0.023**	0.000	73.9%	R
		Caucasians	1.04(0.97-1.11)	0.252	0.010	50.9%	R

F indicates fixed model; R indicates random model

**Table 4:** Summary of results of the meta-analysis from different comparative genetic models in the subgroup analysis by cancer type.

Polymorphism	Genetic comparison	Cancer type	OR(95%CI)	*P*	Test of heterogeneity		Model
					*p* -Value	I^2^	
	AA+TA vs. TT	All	1.04(0.97-1.12)	0.265	0.002,	50.1%	R
		Breast cancer	1.05(0.99-1.10);	0.120	0.462	0.0%	F
		Colorectal cancer	1.04(0.94-1.15)	0.479	0.130	46.9%	F
		Esophageal cancer	0.86(0.44-1.68)	0.652	0.000	90.2%	R
		Others	1.07(0.90-1.26)	0.445	0.007	43.2%	R
	AA vs. TA+TT	All	**1.18(1.06-1.31)**	**0.002**	0.000	56.2%	R
		Breast cancer	**1.20(1.05-1.37)**	**0.007**	0.005	61.5%	R
		Colorectal cancer	1.21(0.76-1.93)	0.416	0.027	67.4%	R
		Esophageal cancer	**1.28(1.08-1.53)**	**0.005**	0.151	47.1%	F
		Others	1.10(0.84-1.44)	0.468	0.015	56.3%	R
	AA vs. TT	All	**1.16(1.01-1.32)**	**0.035**	0.000	55.7%	R
		Breast cancer	**1.22(1.10-1.35**)	**0.000**	0.131	34.6%	F
		Colorectal cancer	1.18(0.72-1.94)	0.501	0.078	56.1%	R
*STK15* F31I		Esophageal cancer	1.02(0.47-2.22)	0.963	0.000	88.6%	R
		Others	1.04(0.77-1.41)	0.794	0.065	44.1%	R
	TA vs. TT	All	1.01(0.95-1.08)	0.745	0.028	37.2%	R
		Breast cancer	1.01(0.96-1.07)	0.667	0.752	0.0%	F
		Colorectal cancer	1.03(0.93-1.15)	0.553	0.313	15.7%	F
		Esophageal cancer	0.78(0.42-1.47)	0.448	0.000	87.5%	R
		Others	1.05(0.94-1.16)	0.392	0.664	0.0%	F
	AA vs. TA	All	**1.18(1.06-1.30)**	**0.001**	0.003	48.4%	R
		Breast cancer	**1.19(1.04-1.36)**	**0.011**	0.011	57.8%	R
		Colorectal cancer	1.25(0.80-1.95)	0.335	0.050	61.7%	R
		Esophageal cancer	**1.32(1.10-1.58)**	**0.003**	0.853	0.0%	F
		Others	1.07(0.83-1.39)	0.591	0.039	49.0%	R
	A vs. T	All	**1.08(1.01-1.14)**	**0.015**	0.000	64.4%	R
		Breast cancer	**1.08(1.01-1.15)**	**0.017**	0.025	52.8%	R
		Colorectal cancer	1.05(0.80-1.38)	0.732	0.008	74.7%	R
		Esophageal cancer	1.00(0.71-1.42)	0.986	0.000	87.9%	R
		Others	1.11(0.95-1.28)	0.180	0.003	64.5%	R

F indicates fixed model; R indicates random model

**Table 5:** Summary of results of the meta-analysis from different comparative genetic models in the breast cancer subgroup analysis by ethnicity.

Polymorphism	Genetic comparison	Population	OR(95%CI)	*P*	Test of heterogeneity		Model
					*p* -Value	I^2^	
	AA+TA vs. TT	All	1.05(0.99-1.10)	0.120	0.462	0.0%	F
		Asians	1.07(0.96-1.20)	0.211	0.482	0.0%	F
		Caucasians	1.04(0.97-1.10)	0.284	0.309	16.3%	F
	AA vs. TA+TT	All	**1.20(1.05-1.37)**	**0.007**	0.005	61.5%	R
		Asians	**1.23(1.00-1.50)**	**0.049**	0.006	75.9%	R
		Caucasians	1.18(0.96-1.44)	0.109	0.055	53.7%	R
	AA vs. TT	All	**1.22(1.10-1.35)**	**0.000**	0.131	34.6%	F
		Asians	**1.21(1.01-1.45)**	**0.037**	0.266	24.3%	F
*STK15* F31I		Caucasians	1.23(0.98-1.54)	0.075	0.081	49.0%	R
	TA vs. TT	All	1.01(0.96-1.07)	0.667	0.752	0.0%	F
		Asians	1.02(0.90-1.14)	0.804	0.492	0.0%	F
		Caucasians	1.01(0.95-1.08)	0.723	0.628	0.0%	F
	AA vs. TA	All	**1.19(1.04-1.36)**	**0.011**	0.011	57.8%	R
		Asians	1.22(0.98-1.52)	0.074	0.005	76.6%	R
		Caucasians	**1.14(1.00-1.29)**	**0.042**	0.136	40.5%	F
	A vs. T	All	**1.08(1.01-1.15)**	**0.017**	0.025	52.8%	R
		Asians	1.15(0.97-1.36)	0.098	0.034	65.5%	R
		Caucasians	1.05(1.00-1.10)	0.069	0.109	44.5%	F

F indicates fixed model; R indicates random model

### Quantitative Synthesis

**Figure 2:**
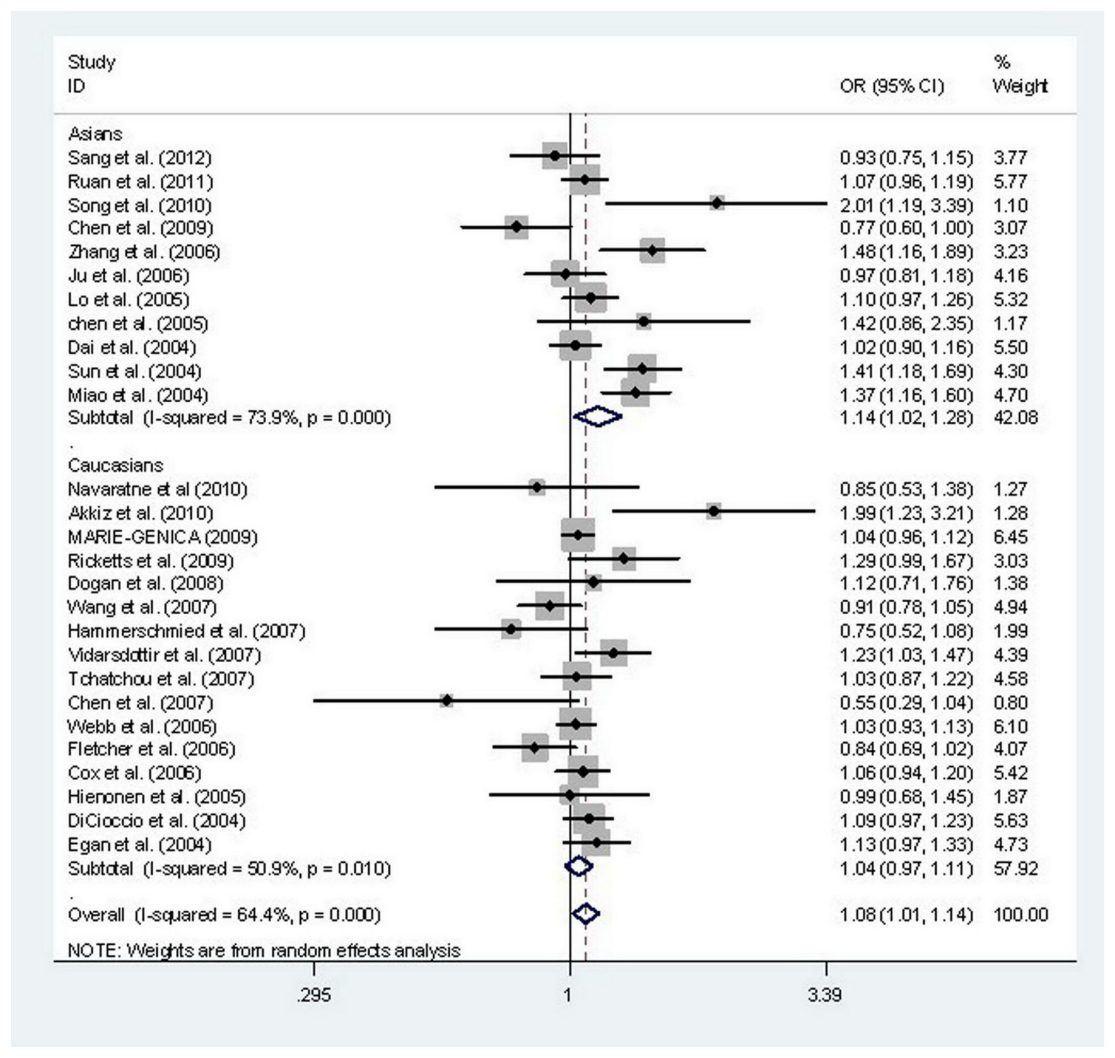
Meta-analysis with a random-effects model for the association between the risk of cancer and the *STK15* F31I polymorphism (A vs. T).

In total, 19,267 multiple cancer cases and 24,359 controls from 27 eligible and original case–control studies were recruited for meta-analysis of the association between the *STK15* F31I polymorphism and cancer risk. Divided by ethnicity, 11 case-control studies were focused on Asian subjects and 16 case-control studies focused on Caucasian subjects. After combining all qualified studies, there was statistical evidence of an association between the *STK15* F31I polymorphism and increased overall cancer risk in four genetic models: AA vs. TA+TT (OR, 1.18; 95% CI, 1.06–1.31; *P*=0.002), AA vs. TT (OR, 1.16; 95% CI, 1.01–1.32; *P*=0.035), AA vs. TA (OR, 1.18; 95% CI, 1.06–1.30; *P*=0.001), and A vs. T (OR, 1.08; 95% CI, 1.01–1.14; *P*=0.015) (Table **3**, Figure **2**). In a stratified analysis by cancer type, there was an increased risk of breast cancer in four genetic models: AA vs. TA+TT (OR, 1.20; 95% CI, 1.05–1.37; *P*=0.007), AA vs. TT (OR, 1.22; 95% CI, 1.10–1.35; *P*=0.000), AA vs. TA (OR, 1.19; 95% CI, 1.04–1.36; *P*=0.011), and A vs. T (OR, 1.08; 95% CI, 1.01–1.15; *P*=0.017) and of esophageal cancer in two genetic model: AA vs. TA+TT (OR, 1.28; 95% CI, 1.08–1.53; *P*=0.005) and AA vs. TA (OR, 1.32; 95% CI, 1.10–1.58; *P*=0.003) (Table **4**). In a stratified analysis by ethnicity, significant increases in cancer risk were observed for Asians, but not Caucasians, for four genetic models: AA vs. TA+TT (OR, 1.27; 95% CI, 1.10–1.47; *P*=0.001), AA vs. TT (OR, 1.26; 95% CI, 1.01–1.56; *P*=0.039), AA vs. TA (OR, 1.28; 95% CI, 1.12–1.47; *P*=0.000) and A vs. T (OR, 1.14; 95% CI, 1.02–1.28; *P*=0.023) (Table **3**). In addition, in a stratified analysis by ethnicity in the breast cancer subgroup, significant increases in cancer risk were observed among Asians for two genetic models: AA vs. TA+TT (OR, 1.23; 95% CI, 1.00–1.50; *P*=0.049) and AA vs. TT (OR, 1.21; 95% CI, 1.01–1.45; *P*=0.037), as well as among Caucasians in one genetic model: AA vs. TA (OR, 1.14; 95% CI, 1.00–1.29; *P*=0.042) (Table **5**)**.**

### Tests for Publication Bias, Sensitivity Analyses, and Heterogeneity

**Figure 3:**
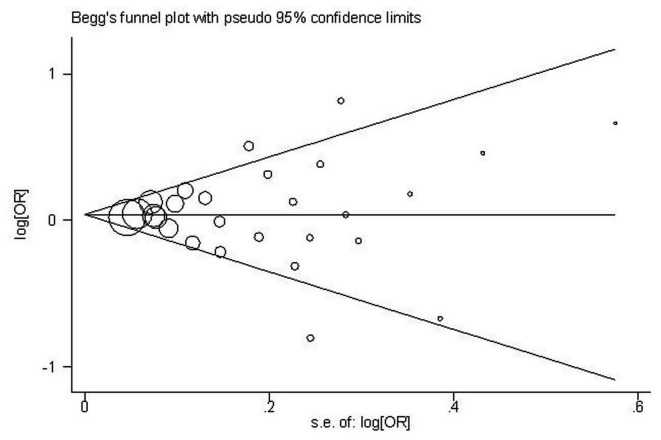
Begg’s funnel plot of meta-analysis of between the *STK15* F31I polymorphism and the risk of cancer in the dominant model.

In this meta-analysis, Begg’s Funnel plot and Egger’s test were both conducted to assess publication bias (Figure **3**). The shape of funnel plot showed the evidence of funnel plot symmetry in all the genetic model. The results indicated that there were no publication bias for overall cancer in current meta-analysis (A vs. T: Begg’s test *P*=0.802, Egger’s test *P*=0.553; AA vs. TT: Begg’s test *P*=1.000, Egger’s test *P*=0.938; TA vs. TT: Begg’s test *P*=0.532, Egger’s test *P*=0.509; AA+TA vs. TT: Begg’s test *P*=0.900, Egger’s test *P*=0.856; AA vs. TT+TA: Begg’s test *P*=0.739, Egger’s test *P*=0.784; AA vs. TA: Begg’s test *P*=0.802, Egger’s test *P*=0.585).

**Figure 4:**
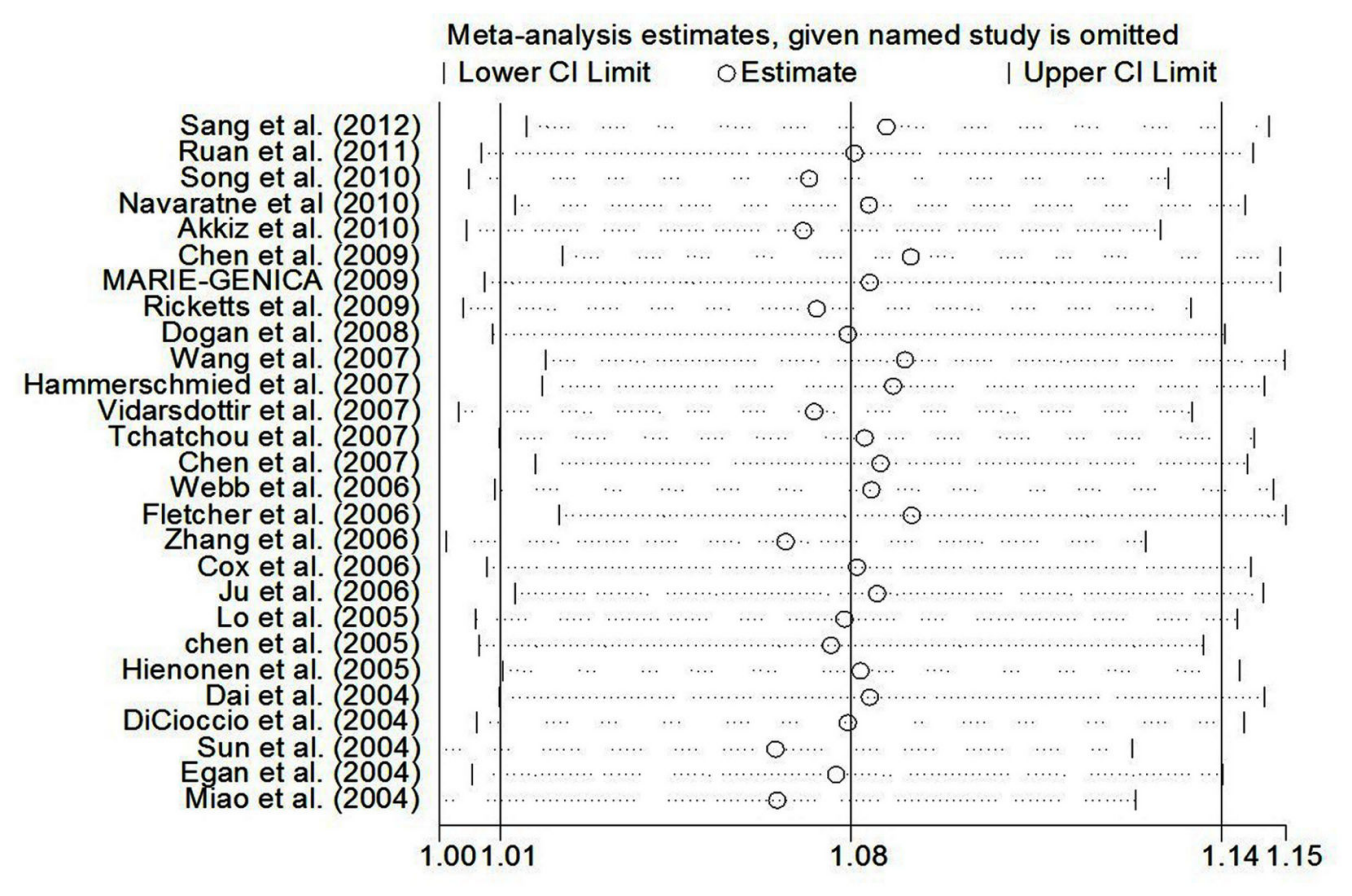
Sensitivity analysis of the influence of A vs. T in overall cancer meta-analysis (random–effects estimates).

**Figure 5:**
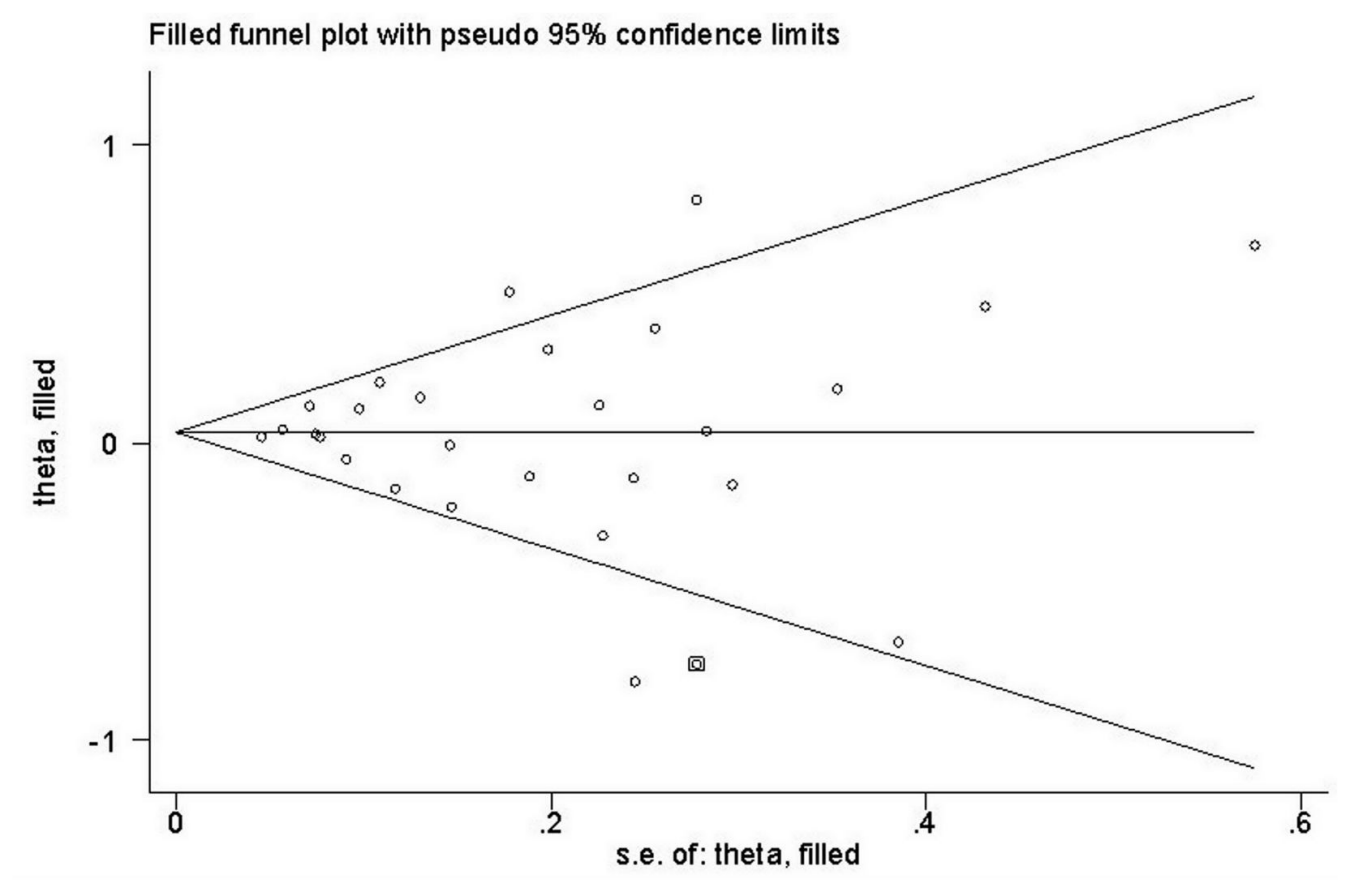
Filled funnel plot of meta-analysis of between the *STK15* F31I polymorphism and the risk of cancer in the dominant model.

Sensitivity analyses were conducted to evaluate the influence of each individual dataset on the pooled OR by deleting each particular dataset dropped at a time. The statistical significances of the overall results did not alter when any individual study was omitted, confirming the stability of the results (Figure **4**). Trim and fill method was also used to perform sensitivity analyses. The findings showed the results of this meta-analysis were reliable (Figure **5**).

**Figure 6:**
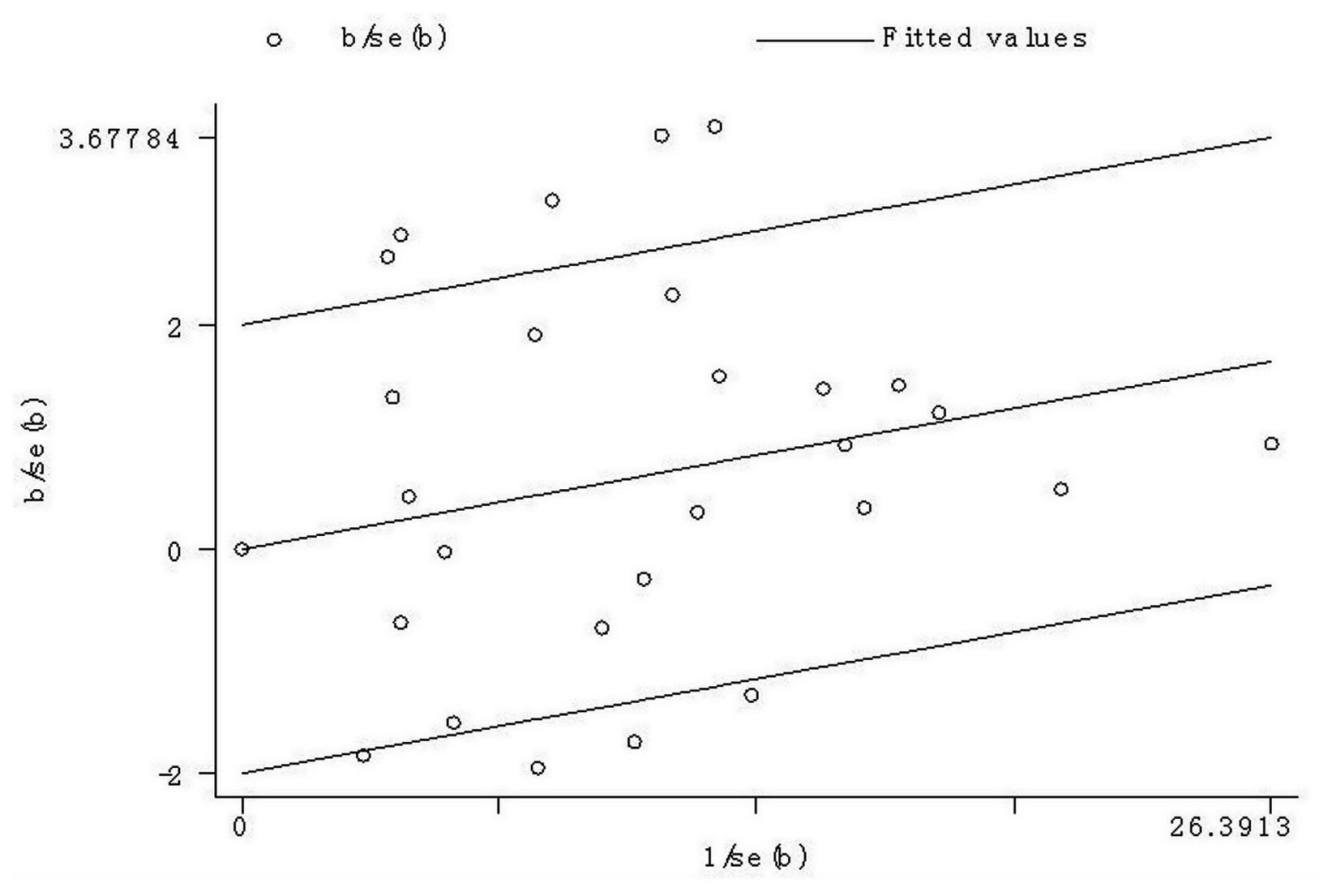
Galbraith radial plot of meta-analysis (A vs. T compare genetic model).

The results showed there were large heterogeneities among the studies enrolled. Because tumor origin and ethnicity can influence the results from meta-analyses, we performed subgroup analyses by cancer type and ethnicity (Table **3** and Table **4**).The results indicated that esophageal cancer, colorectal cancer, Asian population subgroup may contribute to the heterogeneity. As shown in Table **3**, heterogeneity was significant in allele comparison. Galbraith radial plot also was used to analyze the heterogeneity in allele comparison (Figure **6**). The results identified eight outliers which might contribute to the major sources of heterogeneity. Further stratified meta-analysis suggested an association of studies published after 2006, conducted in Chinese population and small sample size design (≤1000 subjects) with more prominent heterogeneity (data not shown).

## Discussion

Accumulating evidence suggests environmental factors, genetic components, and gene–environment interactions play important roles in cancer development and progression [37-42]. Recently, a growing interest in the associations between genetic polymorphisms and cancer risk has led to increasing studies on tumor etiology. Many studies have linked tumor development and progression to the amplification and overexpression of *STK15* in multiple human cancers (such as breast cancer, colorectal cancer, esophageal cancer, as well as other types of cancer) [43-46]. The *STK15* F31I polymorphism has been extensively investigated, and many studies have examined the hypothesis that this polymorphism is relevant to the risk of a variety of cancers; however, the results remain inconclusive and ambiguous. Therefore, we conducted a comprehensive meta-analysis to assess the strength of the association between the *STK15* F31I polymorphism and overall cancer risk, and further performed a stratified analysis by ethnicity and cancer type. This meta-analysis, including 27 case-control studies, identified associations between *STK15* F31I polymorphism and cancer risk. *STK15* F31I polymorphisms (AA vs. TA+TT, AA vs. TT, AA vs. TA, and A vs. T) significantly increased overall cancer risk. In a stratified analysis by cancer type, *STK15* F31I polymorphisms (AA vs. TA+TT, AA vs. TT, AA vs. TA, and A vs. T) were also associated with a significant increase in breast cancer risk and esophageal cancer (AA vs. TA+TT and AA vs. TA). In a stratified analysis by ethnicity, the association of *STK15* F31I polymorphisms was significant in Asians but not Caucasians.

*STK15*, also named Aurora A, BTAK, and AIKI, encodes a serine/threonine kinase that acts as a crucial component in spindle formation, the centrosome maturation process, and proper cytokinesis during mitosis. It is located on chromosome 20q13, a region associated with a number of human cancers [47]. These threonine kinases belong to a family of mitotic kinases that maintain chromosomal stability through phosphorylation. Thus, any severe defects in *STK15*, such as mutations, would lead to drastic genomic instability and trigger apoptosis through cell cycle checkpoint surveillance [19,48]. Consequently, a cell harboring a defective *STK15* may lead to cancer [19]. Our results demonstrate a significant statistical impact of *STK15* F31I polymorphism on cancer risk. The *STK15* F31I polymorphism (T→A), which leads to an amino acid residue substitution at codon 31 phenylalanine (Phe) to isoleucine (Ile), is associated with cellular transformation and dramatically increases chromosomal instability [49]. The *STK15* F31I polymorphism (T→A) variant changes the activity of the *STK15* box 1, leading to an obstruction in p53 binding and the decreased degradation of *STK15* [7]. The stabilized overexpression of *STK15* results in centrosome amplification, improper cytokinesis, chromosomal instability, and the promotion of tumorigenesis [7]. In this meta-analysis, our results demonstrate that the T→A change in *STK15* may lead to *STK15*-triggered elevation of cell centrosome proliferation, cell transformation, and dramatically increased chromosomal instability, which may increase the risk of multiple cancers.

Since the outcomes from meta-analysis can be affected by cancer origins, stratified analysis was conducted according to cancer type for the *STK15* F31I polymorphism. The results demonstrate that the *STK15* F31I polymorphism is associated with an increased risk of breast cancer and esophageal cancer, but not colorectal cancer and other cancers. However, all results should be interpreted with caution. For esophageal cancer, only three case-control studies were recruited in the current meta-analysis, which may restrict statistical power to detect a real influence or generate a fluctuated assessment, large heterogeneities among the studies enrolled in current meta-analysis should also be taken into consideration. More large scale studies are needed to verify these results. Stratified analysis was also performed regarding ethnicity for the *STK15* F31I polymorphism. The *STK15* F31I polymorphism is associated with the risk of cancer in Asians but not Caucasians. This meta-analysis confirmed the mutual effect of genetic diversity and variants in different populations to the risks of various cancers. In addition, cancer risk was affected by genetic and environmental factors on different levels. The possible reason of the conflicting findings among different ethnicities could be that different genetic backgrounds and environmental factors they exposed to may have disproportionate effects on cancer risk. In the future, further investigations with large sample sizes should be conducted to identify these associations, particularly with regard to gene–gene and gene–environment interactions.

Two significant issues should be addressed in this study, that is, heterogeneity and publication bias, which may influence the results of meta-analysis. We don’t detect a significant publication bias in this meta-analysis, suggesting the reliability of our results. Significant heterogeneity was observed between publications for *STK15* F31I polymorphisms. Potential sources of heterogeneity include the publication year, ethnicity, country, cancer type, sample size, and so on. When subgroup analyses were carried out according to ethnicity and cancer type, this heterogeneity was greatly reduced or removed in some subgroups, implying different effects on cancer types and ethnic populations, even for the same polymorphism. And then we performed further subgroup analyses by publication year, country, and sample size. The pooled subgroup analysis of a subset of studies published after 2006, esophageal cancer, Asian population, studies conducted in Chinese population and small sample size, suggested an association with more prominent heterogeneity. The reason might be due to uncontrolled mixed factors, the various susceptibility of cancer in different race or to internal bias in the study design. It is certain that the design of some of the included studies was suboptimal in this meta-analysis. From the forest plot in A vs. T compare genetic model (Figure **2**), one can identify that 8 studies are the main sources of heterogeneity [11,21-23,25,27,33,36]. In some publications, the study design included considerable oversights, for example, some investigations used small sample sizes (≤1000 subjects) [22,23,25,27,33,36]. Publication year may be the source of heterogeneity. Some studies published after 2006 was identified with prominent heterogeneity [22,25,27,33,36]. When come to country origins, studies conducted in Chinese population contribute the major outlier [11,21,23,25,27].

The power of this meta-analysis (α=0.05) was evaluated for each single genetic model using an internet-based Power and Sample Size Calculator (PS, version 3.0, 2009, http://biostat.mc.vanderbilt.edu/twiki/bin/view/Main/PowerSampleSize). The power was 1.000 in four genetic models (AA vs. TA+TT, AA vs. TT, AA vs. TA, and A vs. T), 0.526 in AA+TA vs. TT genetic model, and 0.075 in TA vs. TT genetic model.

 However, there are certain limitations in this study that should be acknowledged. First, large heterogeneity exists in our meta-analysis, which means the results should be interpreted with caution. Second, all recruited case–control studies were from Asians and Caucasians; thus, our results may only be suitable for these populations. Third, only published studies were eligible in this meta-analysis; therefore, some relevant unpublished studies were inevitably missed, which may lead to bias. Fourth, due to the lack of sufficient and uniform information in original case-control studies, data were not stratified by other factors (e.g., age, smoking, alcohol consumption, and other lifestyle factors). Considering the complexity of cancer etiology and the low penetrance cancer susceptibility gene effects from *STK15* F31I SNP, these important environmental factors should not be ignored.

 In summary, this meta-analysis suggests the *STK15* F31I polymorphism represents a low risk factor for cancer, especially in Asians, in breast cancer and esophageal cancer subgroup. In the future, more studies with large sample sizes should be carried out to clarify the association between *STK15* F31I polymorphism and cancer risk, especially for gene–gene and gene–environment interactions.

## Supporting Information

Table S1 **PRISMA checklist, Checklist of items to include when reporting a systematic review or meta-analysis (diagnostic review consisting of cohort studies).**
(DOCX)
